# Supplementary material for: The Expression Characteristics and Function of the RECQ Family in Pan-Cancer
Source: Biomedicines. 2023 Aug 21;11(8):2318. doi: 10.3390/biomedicines11082318 (PMC10452384; doi:10.3390/biomedicines11082318)
Supplement: Supplementary file 1 [file biomedicines-11-02318-s001.zip › biomedicines-2525422-supplementary.pdf]

The expression characteristics and function of the RECQ family in pan-cancer

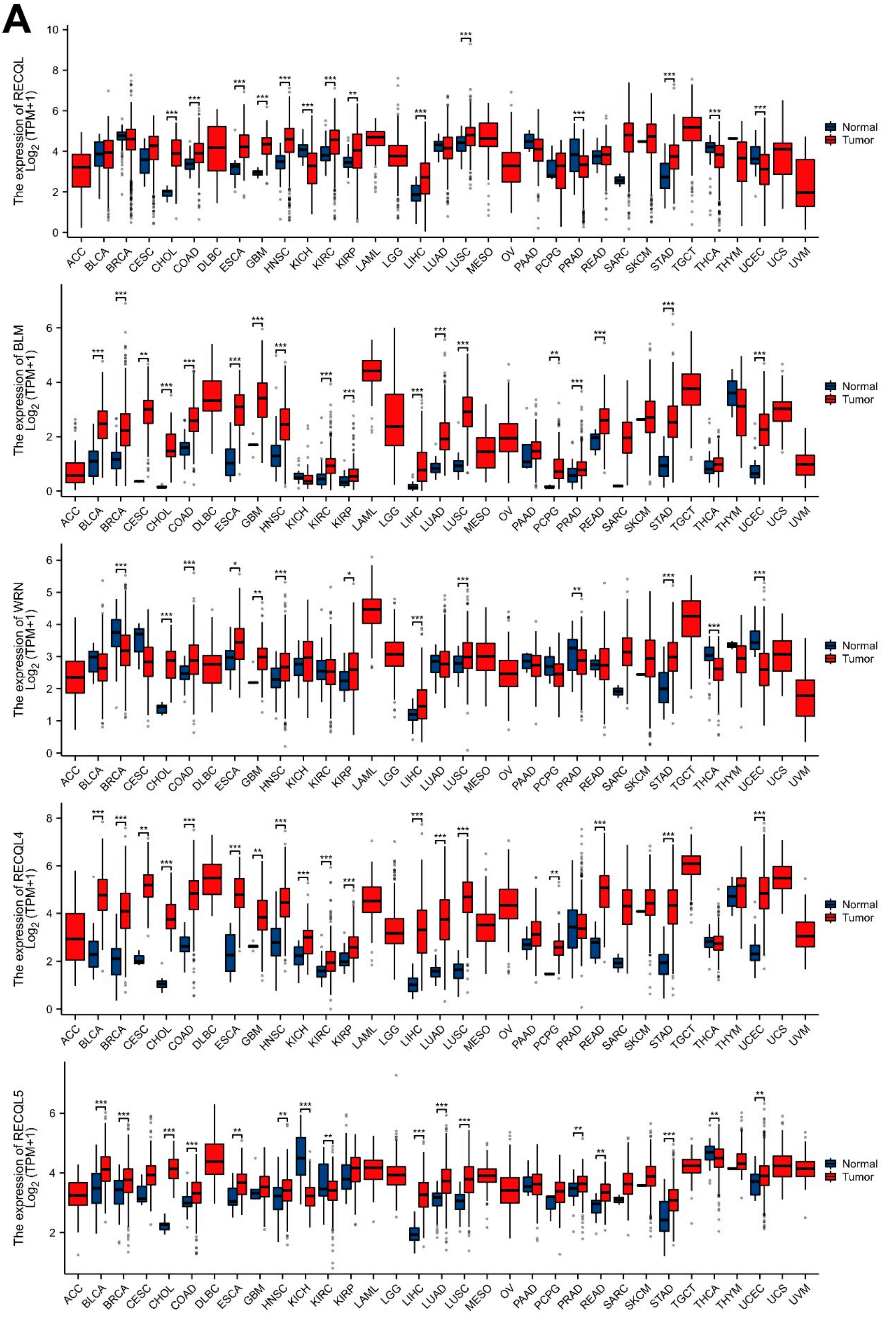

Fig. S1 Expression of RECQ family in tumor and normal tissues. (A) The mRNA expression of RECQs was evaluated across 33 cancers from TCGA. *$p < 0.05$, **$p < 0.01$, ***$p < 0.001$.

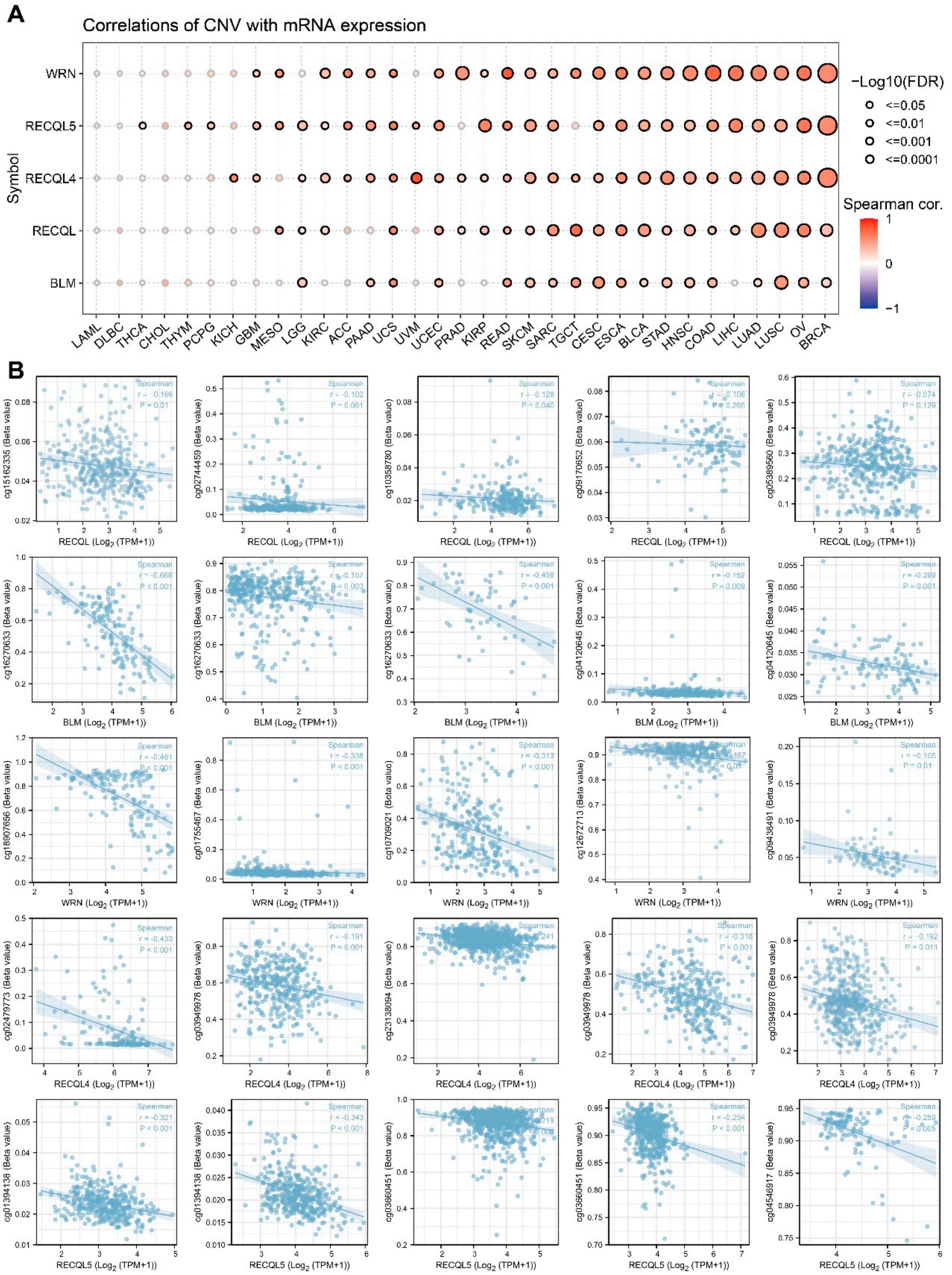

Fig. S2 CNA and DNA methylation of RECQ family in human cancers. (A) The correlation between RECQs mRNA expression and CNV from TCGA. (B) The top 5 negative correlation between RECQs mRNA expression and DNA methylation was demonstrated by TCGA.

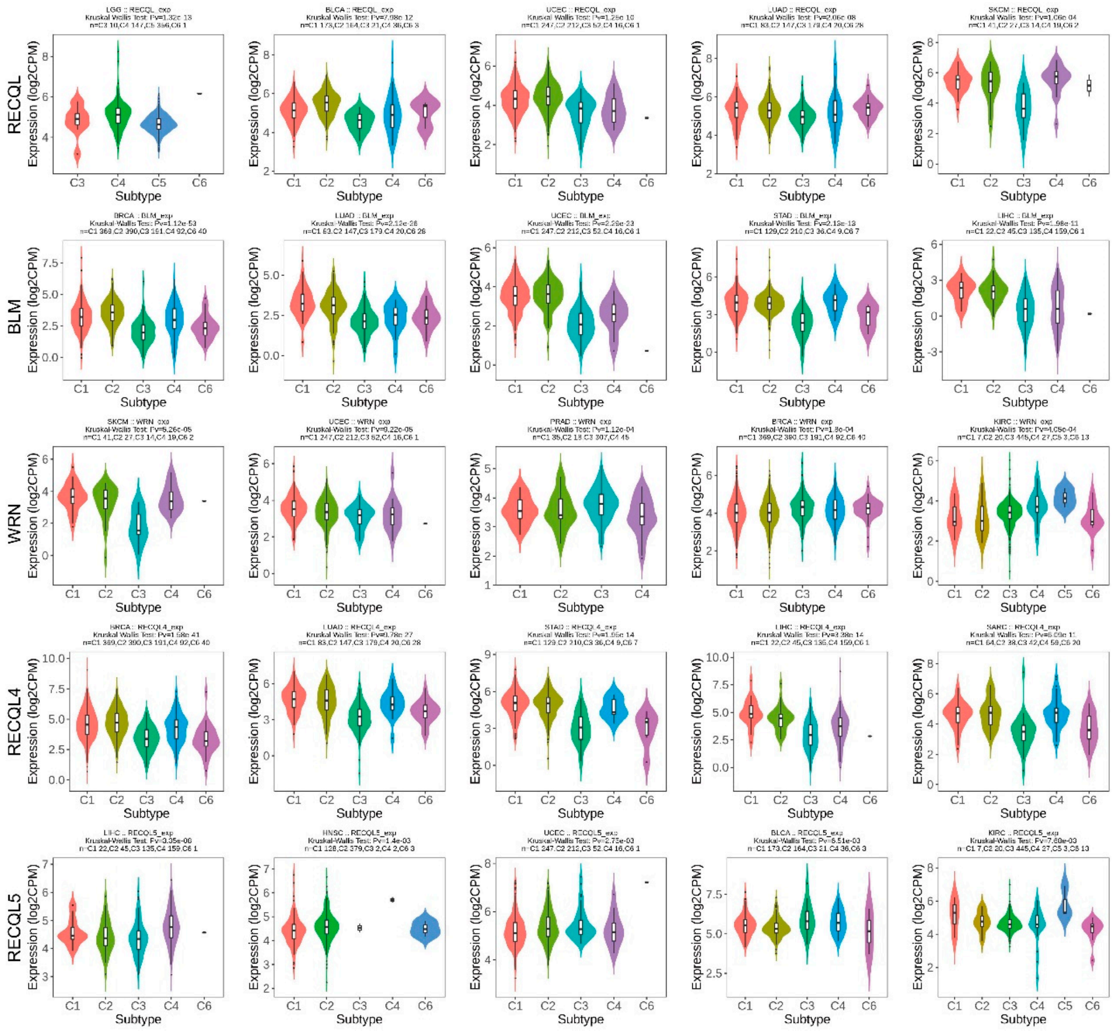

Fig. S3 RECQ mRNA expression in different immune subtypes through TISIDB. Showing the five most statistically significant cancer types for each gene.

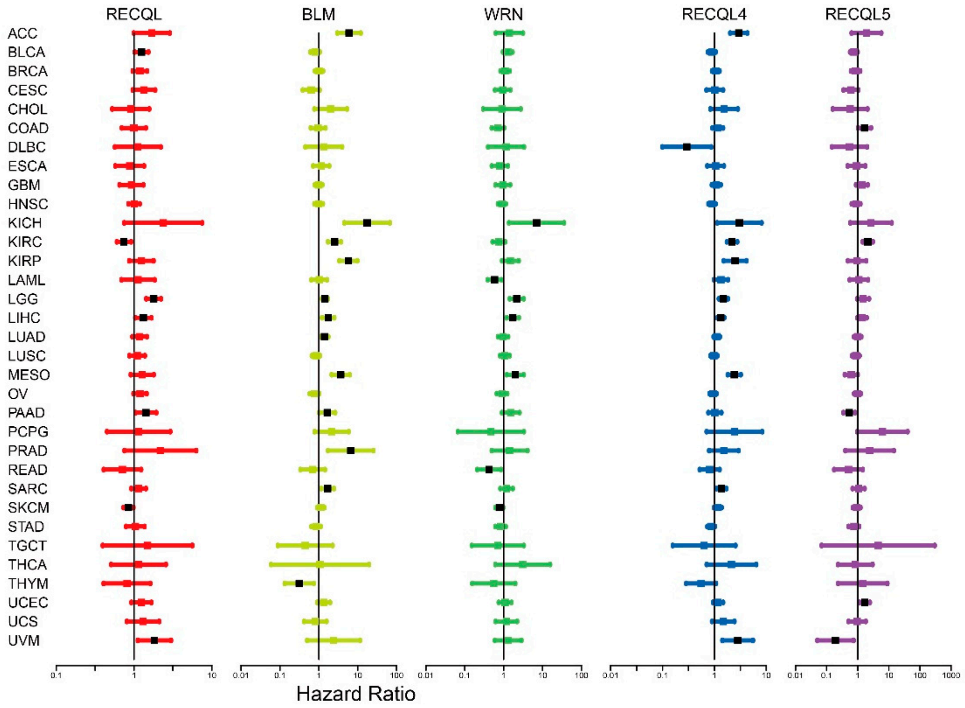

Fig. S4 Overall survival prognosis forest map of RECQ family genes in TCGA. The prominently black labels indicated $p < 0.05$.
